# Comparison of Use of Health Care Services and Spending for Unauthorized Immigrants vs Authorized Immigrants or US Citizens Using a Machine Learning Model

**DOI:** 10.1001/jamanetworkopen.2020.29230

**Published:** 2020-12-11

**Authors:** Fernando A. Wilson, Leah Zallman, José A. Pagán, Alexander N. Ortega, Yang Wang, Moosa Tatar, Jim P. Stimpson

**Affiliations:** 1 Matheson Center for Health Care Studies, University of Utah, Salt Lake City; 2 Department of Economics, University of Utah, Salt Lake City; 3 Department of Population Health Sciences, University of Utah, Salt Lake City; 4 Harvard Medical School, Boston, Massachusetts; 5 Institute for Community Health, Malden, Massachusetts; 6 Cambridge Health Alliance, Cambridge, Massachusetts; 7 Department of Public Health Policy and Management, School of Global Public Health, New York University, New York City; 8 Department of Health Management and Policy, Dornsife School of Public Health, Drexel University, Philadelphia, Pennsylvania; 9 Joseph J. Zilber School of Public Health, University of Wisconsin-Milwaukee

## Abstract

**Importance:**

Knowledge about use of health care services (health care utilization) and expenditures among unauthorized immigrant populations is uncertain because of limitations in ascertaining legal status in population data.

**Objective:**

To examine health care utilization and expenditures that are attributable to unauthorized and authorized immigrants vs US-born individuals.

**Design, Setting, and Participants:**

This cross-sectional study used the data on documentation status from the Los Angeles Family and Neighborhood Survey (LAFANS) to develop a random forest classifier machine learning model. K-fold cross-validation was used to test model performance. The LAFANS is a randomized, multilevel, in-person survey of households residing in Los Angeles County, California, consisting of 2 waves. Wave 1 began in April 2000 and ended in January 2002, and wave 2 began in August 2006 and ended in December 2008. The machine learning model was then applied to a nationally representative database, the 2016-2017 Medical Expenditure Panel Survey (MEPS), to predict health care expenditures and utilization among unauthorized and authorized immigrants and US-born individuals. A generalized linear model analyzed health care expenditures. Logistic regression modeling estimated dichotomous use of emergency department (ED), inpatient, outpatient, and office-based physician visits by immigrant groups with adjusting for confounding factors. Data were analyzed from May 1, 2019, to October 14, 2020.

**Exposures:**

Self-reported immigration status (US-born, authorized, and unauthorized status).

**Main Outcomes and Measures:**

Annual health care expenditures per capita and use of ED, outpatient, inpatient, and office-based physician care.

**Results:**

Of 47 199 MEPS respondents with nonmissing data, 35 079 (74.3%) were US born, 10 816 (22.9%) were authorized immigrants, and 1304 (2.8%) were unauthorized immigrants (51.7% female; mean age, 47.6 [95% CI, 47.4-47.8] years). Compared with authorized immigrants and US-born individuals, unauthorized immigrants were more likely to be aged 18 to 44 years (80.8%), Latino (96.3%), and Spanish speaking (95.2%) and to have less than 12 years of education (53.7%). Half of unauthorized immigrants (47.1%) were uninsured compared with 15.9% of authorized immigrants and 6.0% of US-born individuals. Mean annual health care expenditures per person were $1629 (95% CI, $1330-$1928) for unauthorized immigrants, $3795 (95% CI, $3555-$4035) for authorized immigrants, and $6088 (95% CI, $5935-$6242) for US-born individuals.

**Conclusions and Relevance:**

Contrary to much political discourse in the US, this cross-sectional study found no evidence that unauthorized immigrants are a substantial economic burden on safety net facilities such as EDs. This study illustrates the value of machine learning in the study of unauthorized immigrants using large-scale, secondary databases.

## Key Points

**Question:**

Do unauthorized immigrants have higher health care expenditures and utilization than authorized immigrants or US-born individuals?

**Findings:**

This cross-sectional study used survey data from 47 199 participants and machine learning modeling to predict expenditures and use among unauthorized and authorized immigrants and US-born individuals. Unauthorized immigrants had significantly fewer visits than US-born individuals across settings and were more likely to be uninsured; annual expenditures per person were $1629 for unauthorized immigrants and $3795 for authorized immigrants compared with $6088 for US-born individuals.

**Meaning:**

These findings suggest that unauthorized immigrants are not more likely to use health care and have fewer expenditures than authorized immigrants or US-born individuals.

## Teaser

This cross-sectional study examines health care utilization and expenditures that are attributable to unauthorized immigrants vs authorized immigrants or US-born individuals.

## Introduction

Immigration, particularly unauthorized immigration, continues to be a major topic of social and political debate in the US.^1^ Much of the discourse has centered on the presumption that unauthorized immigrants disproportionately rely on public benefits programs, and this is considered a primary reason that they attempt to migrate to the US.^1^ This assumption has provided partial justification for construction of a security barrier on the southern US border.

These preconceptions have been difficult to refute because limited data are available on unauthorized immigrants. A report by the National Academies of Sciences, Engineering, and Medicine highlighted the importance of insight into the characteristics and behavior of the unauthorized immigrant population to help in the creation of responsive policies.^2^ Unfortunately, direct surveys of this population are difficult and costly owing to deportation risk and, thus, reticence in participating. Few large-scale surveys of unauthorized immigrants have been attempted, particularly surveys of health status and use of health care services (health care utilization). One such survey is the Los Angeles Family and Neighborhood Survey (LAFANS).^3^ The LAFANS is a large, publicly available, secondary database based on a robust randomized sampling design that can be used to study the health and health care utilization of unauthorized immigrants. Other surveys, such as the California Health Interview Survey, the National Health Interview Survey, and the Survey of Income and Program Participation, either restrict access to data on visa status or do not separately identify unauthorized immigrants from those having a legally valid visa.^4,5^

Prior studies have attempted to address this knowledge gap by using either regression-based imputation or a residual approach to impute or predict unauthorized status to respondents in large-scale health care databases.^6,7,8,9,10^ These prior studies suggest that unauthorized immigrants are substantially less likely to use health care services and account for a disproportionately small share of health care expenditures.^6^ In addition to health care, other studies^8^ have used these imputation models to conclude that federal public benefits programs, such as Medicare, would be at higher risk of insolvency in the absence of federal tax revenues generated from unauthorized immigrants. The regression-based imputation models typically use data from organizations, such as the Pew Research Center, that provide a profile of the likely average characteristics of unauthorized immigrants in the US. Unfortunately, because of difficulties in directly studying the unauthorized immigrant population in probability surveys, these data are themselves based on assumptions about the likely factors that are predictive of unauthorized status (eg, place of birth, length of US residency, age, industry employed, income, and other factors).^6,9,10,11^

Alternatively, the residual-based approach to identifying unauthorized respondents classifies respondents as likely to be documented based on meeting specific characteristics, such as arriving in the US before 1980; being a US citizen; receiving Social Security, Medicare, and other public benefits; serving in the US armed forces; working for a government agency; and other factors.^9,10,11^ The remaining respondents who do not meet these characteristics are classified as likely undocumented. The Pew Research Center uses data from the American Community Survey and US Department of Homeland Security. One limitation of this method is that the numbers of potentially unauthorized immigrants may exceed the national residual estimates (total foreign-born population minus lawful immigrant population estimated using Department of Homeland Security data) by 20% to 35%; thus, probabilistic assignment and other adjustment methods are used to assign status and estimate numbers of authorized and unauthorized immigrants.^9,10,11^

Our study provides an alternative methodological approach to study unauthorized immigrant health care outcomes by leveraging the LAFANS database to develop a machine learning-based model for immigrants. Using data on self-reported respondents in LAFANS, the machine learning approach proposed herein is based on a nonparametric classification algorithm that can be used to predict unauthorized status for any other data set with a common set of individual characteristics. We applied this model to identify health care utilization and expenditures among unauthorized and authorized immigrants and US-born individuals using data from the Medical Expenditure Panel Survey (MEPS).

## Methods

For our study, the LAFANS and MEPS data were deidentified, publicly available, and therefore exempt from human subjects protocol as determined by the institutional review board of the University of Utah. This cross-sectional study followed the Strengthening the Reporting of Observational Studies in Epidemiology (STROBE) reporting guideline.

Two sources of data were used for the analysis. First, we used the LAFANS database to construct the machine learning model. The LAFANS is a randomized, multilevel, in-person survey of households residing in Los Angeles County, California. Sampling was based on a stratified random sample of 65 census tracts with oversampling of low-income tracts. Within each tract, households were randomly surveyed. The LAFANS consists of 2 waves. Wave 1 began in April 2000 and ended in January 2002, and wave 2 began in August 2006 and ended in December 2008. Both waves were used for our analysis. Respondents to the LAFANS were asked whether they were born in the US, were a US citizen, were a permanent resident, were granted asylum, or had a visa. Visa holders were further asked whether the visa had expired or was still valid. We classified respondents as unauthorized if they had an expired visa or did not answer “yes” to any of the prior questions. Respondents were classified as authorized immigrants if they were naturalized citizens, permanent residents, or refugees or had an unexpired visa. Respondents who were born in the US were classified as US born. The LAFANS surveyed 1603 US-born individuals and 2179 non–US-born immigrants (771 unauthorized and 1408 authorized immigrants). After listwise deletion, there were 1464 US-born individuals (43.7%) and 1234 authorized (36.9%) and 649 unauthorized (19.4%) immigrants. The LAFANS database was used to build and validate the random forest classifier machine learning model. Details on this procedure and performance metrics are provided in the eMethods and eTable 1 in the Supplement.

Second, we used the 2016-2017 MEPS to examine health care expenditures by immigration status. The year 2017 is the most recent survey year available. The MEPS is a large-scale, nationally representative in-person survey managed by the Agency for Health Care Research and Quality.^12^ Expenditures are measured as reimbursements in dollars by source of payment, including out-of-pocket, private insurance, and Medicare, Medicaid, and other public insurance programs. The analyses are restricted to adults 18 years or older. We applied the random forest classifier machine learning model to predict unauthorized status of MEPS respondents, using the corresponding measures in MEPS that were used in the machine learning model (eMethods in the Supplement). After listwise deletion, our sample included 12 120 non-US-born respondents (3.5% missing) and 35 079 US-born respondents (3.5% missing).

Data were analyzed from May 1, 2019, to October 14, 2020. We stratified health care utilization and expenditures by unauthorized immigrant, authorized immigrant, and US-born status. Health care settings were the emergency department (ED), inpatient nights, outpatient, and physician office.^6^ Total and setting-specific expenditures were top-coded based on the 99th percentile to address outliers. We also examined the percentage of respondents by insurer type and percentage of uncompensated care, which was defined as having any ED or inpatient visit for which the clinician was not reimbursed from any source. This measure is consistent with definitions used in prior research.^6^ Finally, we undertook multivariable regression analyses of immigration status on expenditures and health care utilization, adjusting for sex, age, race/ethnicity, marital status, educational level, language, poverty, insurance, usual source of care, and number of chronic conditions. Race/ethnicity (White non-Latino, Black non-Latino, Latino, and other) was self-reported and included to help ascertain immigration status. Insurance status categories included private insurance, any public insurance (TRICARE, Medicare, Medicaid, State Children’s Health Insurance Program, or other public hospital or physician programs), and uninsured. Usual source of care was based on whether a respondent reported that there is a particular physician’s office, clinic, health center, or other place where he or she usually goes if sick or if advice on health is needed. The number of chronic conditions ranged from 0 to 5 or more. These variables are consistent with measures used in prior research.^6^ Because expenditures are skewed with excess zeros, we used a 2-part model: logistic regression modeling for positive expenditures, and generalized linear modeling with a gamma distribution and log link for health care expenditures, if positive. The modified Park test was used to determine the optimal distribution for the generalized linear model. Logistic regression modeling was used to estimate dichotomous utilization of ED, inpatient, outpatient, and office-based physician visits by immigrant group with adjusting for the above factors. We used χ^2^ tests to determine statistical significance with a 2-sided *P* < .05. Analyses were weighted and conducted using Stata MP, version 16.0 (StataCorp LLC).

## Results

Of 47 199 MEPS respondents with nonmissing data (51.7% female and 48.3% male; mean [SD] age, 47.6 [95% CI, 47.4-47.8] years), 35 079 (74.3%) were US born, 10 816 (22.9%) were authorized immigrants, and 1304 (2.8%) were unauthorized immigrants (Table 1). Compared with other immigrants and US-born individuals, unauthorized immigrants were more likely to be 18 to 44 years of age (80.8% compared with 45.4% and 45.2%, respectively), to be Latino (96.3% compared with 44.0% and 9.4%, respectively), to be Spanish speaking (95.2% compared with 28.2% and 2.1%, respectively), to have less than 12 years of education (53.7% compared with 23.7% and 11.7%, respectively), and to live in poverty (40.6% compared with 12.2% and 10.2%, respectively). Half (49.8%) of unauthorized immigrants do not have a usual source of health care; this compares with less than one-third (30.5%) for authorized immigrants and approximately one-fifth (22.2%) for US-born individuals.

**Table 1.** Descriptive Statistics by Immigration Status

Characteristic	Respondent group^a^			
	All (N = 47 199)	Unauthorized immigrants (n = 1304)	Authorized immigrants (n = 10 816)	US-born individuals (n = 35 079)
Sex				
Male	48.3	49.4	48.3	48.3
Female	51.7	50.6	51.7	51.7
Age, mean (95% CI), y	47.6 (47.4-47.8)	35.3 (34.6-36.1)	48.0 (47.6-48.4)	47.7 (47.5-48.0)
Age, y				
18-44	45.8	80.8	45.4	45.2
45-64	33.5	16.2	36.9	33.2
≥65	20.7	3.0	17.7	21.6
Race/ethnicity				
Non-Latino, White	63.4	0.7	17.4	73.4
Non-Latino, Black	11.7	0	8.4	12.5
Latino	16.1	96.3	44.0	9.4
Other	8.8	3.0	30.2	4.7
Married				
Yes	52.2	52.5	62.6	50.2
No	47.8	47.5	37.4	49.8
Educational level				
Less than high school	14.1	53.7	23.7	11.7
High school	28.7	28.6	22.7	29.9
College	57.2	17.7	53.6	58.4
Spoken language				
Spanish	7.5	95.2	28.2	2.1
English	92.5	4.8	71.8	97.9
Poverty status				
Yes	10.9	40.6	12.2	10.2
No	89.1	59.4	87.8	89.8
Occupation				
Management, business, and financial	11.5	4.2	9.2	11.8
Professional and related	15.4	1.1	15.3	15.6
Service	10.5	20.9	14.7	9.6
Sales and related	5.7	3.0	4.5	6.0
Office and administration	7.2	0.6	6.1	7.6
Farming, fishing, and forestry	0.4	2.0	1.0	0.3
Construction	4.8	11.4	6.5	4.4
Production and transportation	7.6	13.7	9.1	7.2
Other	0.7	1.2	1.1	0.6
Not in labor force	36.2	41.9	32.5	36.9
Usual source of medical care				
No	23.9	49.8	30.5	22.2
Yes	76.1	50.2	69.5	77.8
No. of chronic conditions, mean (95% CI)	1.14 (1.13-1.16)	0.35 (0.30-0.40)	0.82 (0.79-0.85)	1.22 (1.20-1.24)

^a^ Predicted using machine learning–based model. Unless otherwise indicated, data are expressed as weighted percentages of respondents. Data are from the 2016-2017 Medical Expenditure Panel Survey.

Mean annual health care expenditures per person were $1629 (95% CI, $1330-$1928) for unauthorized immigrants, $3795 (95% CI, $3555-$4035) for authorized immigrants, and $6088 (95% CI, $5935-$6242) for US-born individuals (Figure, A). Differences in likelihood of uncompensated care were not statistically significant. Table 2 presents the distribution of mean health care expenditures for respondents who used services across settings. Unauthorized immigrants had significantly lower mean annual inpatient and office-based expenditures ($8589 [95% CI, $5926-$11 251] and $907 [95% CI, $727-$1087], respectively) than authorized immigrants ($ 17 560 [95% CI, $15 378-$19 741] and $1383 [95% CI, $1309-$1457], respectively) or US-born individuals ($18 653 [95% CI, $17 703-$19 603] and $1853 [95% CI, $1808-$1898]). We compared mean expenditures per visit among users across the immigrant groups (eTable 2 in the Supplement). Outpatient and office-based expenditures per visit were lower for unauthorized immigrants vs US-born individuals ($767 [95% CI, $423-$1111] vs $1195 [95% CI, $1120-$1270] for outpatient; $184 [95% CI, $160-$208] vs $239 [95% CI, $234-$245] for office-based). Using data on mean total health care expenditures and weighted numbers of individuals in each group, we estimate that unauthorized immigrants account for $4.2 billion of aggregate national expenditures in 2017; this compares with $155.8 billion for authorized immigrants and $1.3 trillion for US-born individuals (eTable 3 in the Supplement).

**Figure.**
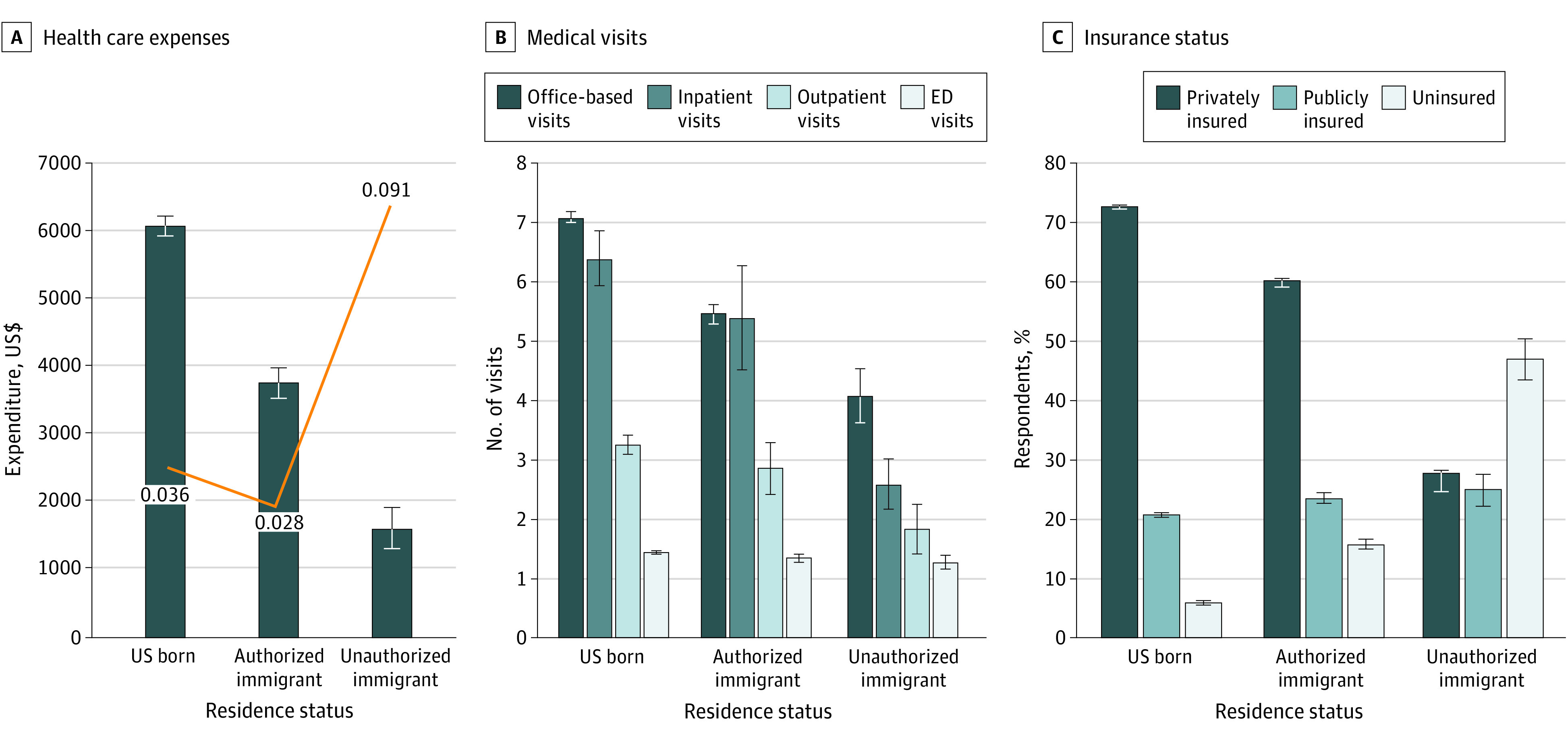
Comparison of Health Care Utilization and Expenses Among Study Groups A, Distribution of mean annual total health care expenses (bars) and percentage with uncompensated care (orange line). B, Mean number of medical visits among users stratified by health care setting. C, Percentage of private, public, or no insurance by residence status. Error bars indicate 95% CIs. Data were obtained from the 2016-2017 Medical Expenditure Panel Survey.

**Table 2.** Annual Expenditures per Person for Respondents Using Health Care by Residence Status^a^

Health care setting	Expenditure by study group, mean (95% CI), $		
	Unauthorized immigrant	Authorized immigrant	US-born individual
ED	1137 (519-1754)	1449 (1306-1591)	1476 (1413-1539)
Inpatient nights	8589 (5926-11 251)	17 560 (15 378-19 741)	18 653 (17 703-19 603)
Outpatient	1536 (435-2636)	2311 (1917-2706)	2669 (2527-2811)
Office based	907 (727-1087)	1383 (1309-1457)	1853 (1808-1898)

Abbreviation: ED, emergency department.

^a^ Data are from the 2016-2017 Medical Expenditure Panel Survey.

Among respondents who used care at least once, unauthorized immigrants had fewer visits than authorized immigrants or US-born individuals (Figure, B). For example, among respondents reporting at least 1 office-based visit, US-born individuals had a mean of 7.1 (95% CI, 7.0-7.2) visits per year vs 4.1 (95% CI, 3.6-4.5) visits for unauthorized immigrants.

Figure, C, reports type of insurance by immigration status. Nearly three-quarters (73.1%) of US-born individuals were privately insured compared with a little more than one-quarter (27.9%) of unauthorized immigrants. Unauthorized immigrants were not more likely to receive public insurance than authorized immigrants but were slightly more likely to receive public insurance than US-born individuals (27.9% vs 20.8%). Nearly one-half (47.1%) of unauthorized immigrants were predicted to be uninsured—substantially higher than rates for authorized immigrants (15.9%) and US-born individuals (6.0%).

Generalized linear models and logistic regression models examined immigration status with health care expenses and dichotomous measures of having any health care expenditure and any health care utilization (ED, inpatient, outpatient, or office based) (Table 3). Unauthorized immigrants were more likely than US-born respondents to report having any health care expenditures (adjusted odds ratio [AOR], 1.24; 95% CI, 1.01-1.52), but there was no statistically significant difference for authorized immigrants. Among individuals reporting positive health care expenditures, expenditures were 29% smaller (coefficient, −0.29; 95% CI, −0.54 to −0.04) for unauthorized immigrants than US-born individuals; for authorized immigrants, expenditures were 17% smaller (coefficient, −0.17; 95% CI, −0.25 to −0.09). Authorized immigrants were less likely to have any health care utilization than US-born individuals, except for office-based visits (AOR, 1.12; 95% CI, 0.91-1.38), which was statistically insignificant. Differences in utilization between unauthorized immigrants and US-born individuals were not statistically significant after adjusting for confounding factors. eTable 4 in the Supplement provides generalized linear modeling-adjusted annual health care expenditures per person by immigration status. Adjusting for confounding factors, no statistically significant difference in expenditures was found between unauthorized and authorized immigrants ($4105 [95% CI, $3094-$5115] and $4622 [95% CI, $4288-$4955], respectively). However, both expenditures were significantly less than that for US-born individuals ($5490 [95% CI, $5338-$5641]).

**Table 3.** Generalized Linear and Logistic Regression Modeling of Total Health Care Expenditures and Utilization^a^

Measure	Any health care expense, AOR (95% CI)^b^	Health care expenses if positive, coefficient (95% CI)^c^	Visit type, AOR (95% CI)^b^			
			Any ED	Any inpatient nights	Any outpatient	Any office based
Residence status						
US-born	1 [Reference]	1 [Reference]	1 [Reference]	1 [Reference]	1 [Reference]	1 [Reference]
Authorized immigrant	0.94 (0.85 to 1.05)	−0.17 (−0.25 to −0.09)	0.71 (0.64 to 0.80)	0.69 (0.58 to 0.81)	0.81 (0.72 to 0.90)	0.97 (0.88 to 1.06)
Unauthorized immigrant	1.24 (1.01 to 1.52)	−0.29 (−0.54 to −0.04)	0.77 (0.59 to 1.02)	0.95 (0.66 to 1.37)	1.35 (0.98 to 1.85)	1.12 (0.91 to 1.38)
Sex						
Female	1 [Reference]	1 [Reference]	1 [Reference]	1 [Reference]	1 [Reference]	1 [Reference]
Male	0.46 (0.43 to 0.50)	−0.22 (−0.27 to −0.17)	0.75 (0.70 to 0.81)	0.60 (0.54 to 0.65)	0.65 (0.61 to 0.70)	0.49 (0.46 to 0.52)
Age, y						
18-44	1 [Reference]	1 [Reference]	1 [Reference]	1 [Reference]	1 [Reference]	1 [Reference]
45-64	1.23 (1.13 to 1.35)	0.17 (0.11 to 0.24)	0.78 (0.71 to 0.85)	0.68 (0.60 to 0.77)	1.52 (1.40 to 1.65)	1.30 (1.20 to 1.40)
≥65	2.05 (1.73 to 2.43)	0.25 (0.18 to 0.32)	0.78 (0.70 to 0.87)	1.03 (0.90 to 1.18)	1.72 (1.56 to 1.90)	2.10 (1.85 to 2.37)
Race/ethnicity						
Non-Latino						
White	1 [Reference]	1 [Reference]	1 [Reference]	1 [Reference]	1 [Reference]	1 [Reference]
Black	0.53 (0.48 to 0.58)	−0.19 (−0.27 to −0.12)	1.18 (1.08 to 1.28)	0.94 (0.83 to 1.06)	0.69 (0.63 to 0.75)	0.59 (0.54 to 0.64)
Latino	0.78 (0.70 to 0.88)	−0.07 (−0.16 to 0.02)	1.05 (0.94 to 1.18)	0.97 (0.82 to 1.14)	0.67 (0.59 to 0.75)	0.79 (0.72 to 0.88)
Other	0.72 (0.63 to 0.82)	−0.13 (−0.23 to −0.04)	0.98 (0.85 to 1.13)	0.98 (0.81 to 1.18)	0.79 (0.70 to 0.90)	0.74 (0.66 to 0.82)
Marital status						
Not married	1 [Reference]	1 [Reference]	1 [Reference]	1 [Reference]	1 [Reference]	1 [Reference]
Married	1.08 (0.99 to 1.17)	0.05 (−0.004 to 0.10)	0.84 (0.78 to 0.90)	1.16 (1.06 to 1.28)	1.05 (0.98 to 1.12)	1.05 (0.98 to 1.12)
Educational level						
Less than high school	1 [Reference]	1 [Reference]	1 [Reference]	1 [Reference]	1 [Reference]	1 [Reference]
High school	0.99 (0.89 to 1.10)	−0.02 (−0.11 to 0.06)	1.04 (0.95 to 1.15)	0.97 (0.86 to 1.11)	1.34 (1.21 to 1.49)	1.05 (0.95 to 1.15)
College	1.66 (1.49 to 1.85)	0.08 (−0.003 to 0.16)	0.86 (0.78 to 0.95)	0.95 (0.84 to 1.08)	1.36 (1.23 to 1.50)	1.57 (1.43 to 1.73)
Language						
English or other	1 [Reference]	1 [Reference]	1 [Reference]	1 [Reference]	1 [Reference]	1 [Reference]
Spanish	0.60 (0.52 to 0.68)	−0.10 (−0.23 to 0.04)	0.93 (0.81 to 1.08)	0.97 (0.79 to 1.20)	0.74 (0.63 to 0.88)	0.70 (0.63 to 0.79)
Poverty status						
No	1 [Reference]	1 [Reference]	1 [Reference]	1 [Reference]	1 [Reference]	1 [Reference]
Yes	0.96 (0.86 to 1.06)	0.18 (0.10 to 0.26)	1.34 (1.23 to 1.47)	1.54 (1.37 to 1.74)	1.11 (1.0 to 1.22)	0.96 (0.87 to 1.05)
Insurance						
Private	1 [Reference]	1 [Reference]	1 [Reference]	1 [Reference]	1 [Reference]	1 [Reference]
Public	0.79 (0.71 to 0.88)	0.13 (0.07 to 0.19)	1.53 (1.40 to 1.66)	1.47 (1.31 to 1.64)	1.05 (0.97 to 1.14)	0.86 (0.78 to 0.93)
Uninsured	0.33 (0.30 to 0.37)	−0.41 (−0.57 to −0.25)	1.05 (0.91 to 1.20)	0.70 (0.55 to 0.89)	0.50 (0.42 to 0.60)	0.36 (0.33 to 0.40)
Usual source of care						
No	1 [Reference]	1 [Reference]	1 [Reference]	1 [Reference]	1 [Reference]	1 [Reference]
Yes	3.19 (2.94 to 3.45)	0.26 (0.18 to 0.34)	1.23 (1.12 to 1.35)	1.20 (1.05 to 1.37)	2.20 (1.99 to 2.44)	3.14 (2.93 to 3.36)
No. of chronic conditions	2.20 (2.06 to 2.34)	0.35 (0.33 to 0.37)	1.45 (1.41 to 1.49)	1.57 (1.52 to 1.63)	1.31 (1.27 to 1.34)	1.78 (1.71 to 1.85)

Abbreviations: AOR, adjusted odds ratio; ED, emergency department.

^a^ Data are from the 2016-2017 Medical Expenditure Panel Survey.

^b^ Results from logistic regression model.

^c^ Results from generalized linear model regression with gamma distribution and log link.

## Discussion

Our study uses a machine learning approach based on population-based survey data of immigrants to provide insight into differences in health care utilization and expenditures among unauthorized and authorized immigrants and US-born individuals. Our findings suggest that unauthorized and authorized immigrants have significantly lower health care expenditures than US-born individuals. Total mean health care expenditures are more than $4400 less for unauthorized immigrants than US-born individuals per year. Despite our finding that unauthorized immigrants are 8 times more likely to be uninsured than US-born individuals, rates of uncompensated care are not statistically significant between unauthorized immigrants and US-born individuals.

Recent public policy debates in the US have focused on decreasing unauthorized immigration from Mexico, and some policy makers argue that unauthorized immigrants are a net drain on federal and state benefits programs. Prior research has suggested a more complex story than this. One study found that the US Medicare Trust Fund would reach insolvency at an earlier date than anticipated in the absence of substantial contributions being made to the fund by unauthorized immigrants.^8^ Our findings imply that there is also little evidence that unauthorized immigrants are leading to overcrowded EDs and threatening the financial viability of hospitals owing to high rates of uncompensated care.

Our study confirms prior research on immigrants and health care expenditures, which found generally lower expenditures for immigrants than US-born individuals.^13,14,15,16,17,18^ Reasons for these findings are unclear, but may be associated with the Latino epidemiological paradox.^19^ For example, prior research by immigration scholars suggests that immigrants may be healthier than nonimmigrants.^20,21^ However, unauthorized immigrants experience substantial legal and economic barriers to accessing health care in the US.^22^ In addition to risks of arrest and deportation, unauthorized immigrants are largely excluded from state benefits and do not qualify to receive any federally funded benefits programs owing to the 1996 Personal Responsibility and Work Opportunity Reconciliation Act. Unauthorized immigrants have also been excluded from participation in the health insurance exchanges established under the Patient Protection and Affordable Care Act, even if they are willing to forego health insurance subsidies. As a result of these barriers, immigrants are less likely to have a usual source of care other than an ED.^23^ This is reflected in our data, with half of unauthorized immigrants lacking a usual source of care. Unauthorized immigrants also have substantially higher rates of being uninsured than authorized immigrants and US-born individuals in our data, with half of unauthorized immigrants lacking health insurance, consistent with prior research.^22,23,24,25^ Although implementation of the Patient Protection and Affordable Care Act may have helped decrease the uninsured population, these benefits were smaller for noncitizen immigrants than for naturalized citizens or US-born individuals.^26^

However, the epidemiological paradox does not necessarily mean that our finding of lower utilization of health care for immigrants than US-born individuals is desirable. Given immigrant communities’ vulnerability and barriers to accessing care, it is possible that some of their health care needs are not being met. There is evidence that much of the epidemiological paradox may be the result of undiagnosed disease among immigrants, and there is uncertainty over whether the paradox applies to unauthorized immigrants.^27^ For example, results from one study^28^ indicate that systolic blood pressure and odds of hypertension are not lower for unauthorized immigrants than for US-born individuals. We expect that rates of undiagnosed disease are likely to be especially high among unauthorized immigrants, resulting in high health care treatment costs in the future, but further research is needed.

Under the 1986 Emergency Medical Treatment and Labor Act, it is illegal for hospitals to turn away patients who present at an ED regardless of health insurance coverage status. Thus, it is commonly assumed by many people in the debate over immigration that unauthorized immigrants rely on EDs as their primary source of health care. Interestingly, despite high rates of no insurance and poverty among the unauthorized immigrants, our data do not indicate an excessive use of ED services. Furthermore, differences in uncompensated rates were not statistically significant across immigrant groups in our study. It is surprising that the differences are not more pronounced because of the exceedingly high rate of uninsured individuals (47.1%) in unauthorized immigrant communities, suggesting an area of future study.

The performance of the machine learning approach is contingent on accuracy of self-reported immigration status in the LAFANS. A strength of the LAFANS is that respondents are not directly asked about their unauthorized status, but rather this is determined indirectly based on negative responses to questions about citizenship, permanent residency, and visas held. Prior research has reported that the profile of unauthorized immigrants from the LAFANS is similar to that found using the residual method.^5^ Furthermore, using our machine learning approach, the characteristics of unauthorized immigrants are generally consistent with those in other sources, although there are substantial limitations with all attempts to characterize unauthorized immigrants (eMethods in the Supplement). It should be noted that the National Academies of Sciences, Engineering, and Medicine specifically mentioned LAFANS in recommending that similar questions on legal status be added to other widely used databases, such as the National Health Interview Survey, the National Educational Longitudinal Survey, and the National Health and Nutrition Examination Survey.^2^

### Limitations

This study has some limitations. Although the machine learning model used in our study has an accuracy rate of 91%, approximately 9% of immigrants will be incorrectly classified. The machine learning methodology is also sensitive to the quality and generalizability of the data provided for learning. The LAFANS only surveyed the Los Angeles area, and results may not be generalizable. Furthermore, LAFANS data were collected more than 10 years ago, and characteristics of unauthorized immigrants may have changed since then. Unauthorized immigrants may also be underrepresented in LAFANS owing to concerns over participating in the survey. However, the unique strengths of LAFANS in identifying characteristics predictive of unauthorized status may offset these limitations. By extension, our machine learning methodology requires measures that were collected in common between LAFANS and MEPS. Thus, there may be measures that are predictive of unauthorized status in LAFANS that were excluded because comparable measures did not exist in MEPS. Finally, our sample size of unauthorized immigrants is modest (1304 unauthorized immigrants) and thus may also limit the generalizability of results.

## Conclusions

The unauthorized immigrant population in the US experiences substantial economic and legal vulnerabilities that make it one of the most difficult communities to study. Consequently, our knowledge of the implications of these vulnerabilities on health care access and services is limited. Research is critical to understand better the health status of this vulnerable population and to design effective interventions and policies that can address persistent gaps in access to care. To address this knowledge gap, our study used machine learning to examine health care access among unauthorized immigrants. Contrary to much political discourse in the US, we find no evidence that unauthorized immigrants pose a substantial economic burden on the health care delivery system in the US.

## Notes

Supplement. **eMethods.** Machine Learning Model and Analysis **eTable 1.** Model Performance Metrics of Random Forest Classifier, Decision Tree Classifier, and Boosted Machine Learning Models Using K-fold Cross-validation, MEPS 2016-2017 **eTable 2.** Mean Expenditures per Visit for Respondents Using Health Care by Immigration Status, MEPS 2016-2017 **eTable 3.** Unweighted and Weighted Numbers and Estimated Aggregate National Health Care Expenditures for Each Immigrant Group **eTable 4.** Generalized Linear Model Adjusted Total Health Care Expenditures by Immigration Status
