# Supplementary material for: Comparison of Use of Health Care Services and Spending for Unauthorized Immigrants vs Authorized Immigrants or US Citizens Using a Machine Learning Model
Source: JAMA Netw Open. 2020 Dec 11;3(12):e2029230. doi: 10.1001/jamanetworkopen.2020.29230 (PMC7733155; doi:10.1001/jamanetworkopen.2020.29230)
Supplement: Supplement. — eMethods. Machine Learning Model and Analysis eTable 1. Model Performance Metrics of Random Forest Classifier, Decision Tree Classifier, and Boosted Machine Learning Models Using K-fold Cross-validation, MEPS 2016-2017 eTable 2. Mean Expenditures per Visit for Respondents Using Health Care by Immigration Status, MEPS 2016-2017 eTable 3. Unweighted and Weighted Numbers and Estimated Aggregate National Health Care Expenditures for Each Immigrant Group eTable 4. Generalized Linear Model Adjusted Total Health Care Expenditures by Immigration Status [file jamanetwopen-e2029230-s001.pdf]

Supplementary Online Content

Wilson FA, Zallman L, Pagán JA, et al. Comparison of use of health care services and spending for unauthorized immigrants vs authorized immigrants or US citizens using a machine learning model. *JAMA Netw Open*. 2020;3(12):e2029230. doi:10.1001/jamanetworkopen.2020.29230

eMethods. Machine Learning Model and Analysis

eTable 1. Model Performance Metrics of Random Forest Classifier, Decision Tree Classifier, and Boosted Machine Learning Models Using K-fold Cross-validation, MEPS 2016-2017

eTable 2. Mean Expenditures per Visit for Respondents Using Health Care by Immigration Status, MEPS 2016-2017

eTable 3. Unweighted and Weighted Numbers and Estimated Aggregate National Health Care Expenditures for Each Immigrant Group

eTable 4. Generalized Linear Model Adjusted Total Health Care Expenditures by Immigration Status

This supplementary material has been provided by the authors to give readers additional information about their work.

©2020 Wilson FA et al. *JAMA Network Open*.

eMethods. Machine Learning Model and Analysis

The machine learning procedure consisted of the following steps. Respondents to the L.A.FANS were asked whether they were born in the US, were a US citizen, were a permanent resident (possessed a green card), were granted asylum, or had a visa to stay in the US. For visa holders, they were further asked whether the visa had expired or was still valid. We classified respondents as unauthorized if they had an expired visa or did not answer "yes" to any of the prior questions. Respondents were classified as authorized immigrants if they were naturalized citizens, permanent residents, refugees, or had an unexpired visa. Respondents who were born in the US were classified as US-born individuals. The L.A.FANS surveyed 1,603 US-born and 2,179 non-US-born immigrants (771 unauthorized and 1,408 authorized immigrants). After listwise deletion, there were 1,464 (43.7%) US-born and 1,234 (36.9%) authorized and 649 (19.4%) unauthorized immigrants.

Second, in predicting unauthorized versus authorized status, the ML model used the following variables: sex, age in years, race/ethnicity, marital status (married versus not married), education status (less than high school, high school, college), language of interview (Spanish versus English/Other), poverty status, and occupation. Race/ethnicity included white, black, Latino or Other. Poverty status was based on having household income above versus below 100% of the federal poverty line. Occupation included the following categories: management business & financial, professional & related, service, sales & related occupation, office & administration, farming fishing & forestry, construction extraction & maintenance, production transportation & material moving, other, and not in labor force. These variables were selected based on a review of measures that were commonly defined across the L.A.FANS and MEPS databases (see below).

Third, using the above data from L.A.FANS, we applied a random forest classifier ML model using the L.A.FANS dataset (3,348 observations) described above. Python 3.7 was used for programming using the scikit-learn machine learning library. Scikit-learn is a powerful library of tools to undertake machine learning analyses using Python. In order to test model performance, we split-tested the model using K-fold cross-validation (CV). For K-fold CV, the L.A.FANS dataset was subdivided into 5 folds, which were used as testing sets for the ML model. K-fold CV suggested that the model had 90.5% accuracy (standard deviation (SD) = 2.1%) and 93.9% specificity (SD = 2.3) in predicting unauthorized versus authorized immigration status (eTable 1). Sensitivity (76.1%, SD = 6.2) and precision (75.7%, SD = 4.7) were comparable. The F-measure was 75.7 (SD = 3.6).

Finally, we applied the random forest classifier ML model to predict unauthorized status of MEPS respondents, using the corresponding measures in MEPS that were used in the ML model. The 2016-2017 MEPS includes 12,554 non-US-born respondents. Respondents with missing data for the above variables were listwise deleted, which resulted in 12,120 non-US-born respondents (3.5% missing). A total of 36,341 respondents were US-born. Listwise deletion resulted in 35,079 US-born respondents (3.5% missing).

Sensitivity analyses included examining alternative machine learning models. eTable 1 compares accuracy, specific, sensitivity, precision and F-measures for random forest classifier, decision tree classifier and boosted classifier (AdaBoost) models. These are widely used non-parametric machine learning approaches based on classifying observations into two groups. The random forest classifier model has improved metrics than the other approaches; for example, its F-measure is 75.7 compared to 74.5 for the boosted model and 73.0 for the decision tree classifier. However, differences are not statistically significant.

We compared percent unauthorized and their socio-demographic characteristics from our ML-based approach with corresponding estimates from the Pew Research Center.[1,6-8,13] The ML approach is more conservative in classifying respondents as unauthorized, accounting for 1.2% (2.6% unweighted) of MEPS respondents versus 3.2% for the latest 2017 data reported by the Pew Research Center.[1] Reasons for this difference are unclear, and it may be reasonable to assume that the true percentage lies within this range.

In our opinion, there is no authoritative source of data on the unauthorized, thus, imputation has been an important technique for researchers studying this population. There is a general lack of detailed information on the unauthorized and substantial limitations associated with all attempts to characterize unauthorized immigrants. A comparison of our estimates using

[1] Krogstad JM, Passel JS, Cohn D. 5 Facts about 1 illegal immigration in the US. Pew Research Center. 2 Published June 12, 2019. Accessed May 20, 2020. 3 https://www.pewresearch.org/fact-tank/2019/06/12/5-4 facts-about-illegal-immigration-in-the-u-s/

©2020 Wilson FA et al. *JAMA Network Open*.

machine learning with alternative estimates of the unauthorized population should be interpreted in the context of limitations associated with all the approaches used. That being said, we will compare our characteristics of the unauthorized to two other well-known sources.

The first source, a 2018 report by the Pew Research Center (PRC), uses the residual approach with data from the year 2016. This approach starts with the weighted number of the total foreign-born population in the US and then uses US Department of Homeland Security data on lawful immigrant admissions to estimate the total number of unauthorized immigrants in the US. Afterward, they probabilistically assign unauthorized immigration status using either the American Community Survey or the Current Population Survey based on: "individual's demographic, social, economic, geographic and family characteristics in numbers that agree with the initial residual estimates for the estimated lawful immigrant and unauthorized immigrant populations in the survey".[2]

The second source is the Migration Policy Institute (MPI). Their methodology imputes unauthorized status based on the 2012-2016 American Community Survey and using the 2008 Survey of Income and Program Participation (SIPP).[3]

In the table below, our machine learning (ML) approach suggests that 49.4% of unauthorized immigrants are male compared to 54% for the PRC and 53% for the MPI approaches. Both the PRC and ML approaches suggest unauthorized immigrants skew to younger ages with 80.8% and 69% aged 18-44 for ML and PRC, respectively. Approximately half of unauthorized immigrants have less than high school education (53.7% for ML; 47.0% for MPI). However, the ML approach suggests significantly higher rates of poverty (40.6%) versus 28% for MPI.

Demographics	Machine Learning 2017-18 data	Pew Research Center 2016 data	Migration Policy Institute 2012-16 data
**Sex**			
**Male**	49.4	54.0	53.0
**Female**	50.6	46.0	47.0
**Age**			
**18-44**	80.8	69.0	n/a
**45-64**	16.2	30.0	n/a
**65 and over**	3.0	1.0	n/a
**Education**			
**Less than high school**	53.7	n/a	47.0
**In poverty**	40.6	n/a	28.0

The US Census Bureau does not provide information on the number of unauthorized immigrants residing in the US. However, it reports 44.7 million foreign-born residents in 2019. This compares to 43.1 million in 2017 in our data after weighting (refer to table below).

Group	Weighted N in 2017 (in millions)	2019 US Census Bureau* (in millions)
**US-born**	204.4	205.9
**Authorized**	40.1	
**Unauthorized**	3.0	
**Foreign-born**	43.1	44.7
**Total**	247.5	250.6

* US Census Bureau American Community Survey estimates for 2019.

[2] Pew Research Center. U.S. Unauthorized Immigrant Total Dips to Lowest Level in a Decade. November 27, 2018. For a more complete review of the Pew Research Center methodology, please visit: https://www.pewresearch.org/hispanic/2018/11/27/unauthorized-immigration-estimate-methodology/
[3] Refer to: https://www.migrationpolicy.org/programs/us-immigration-policy-program-data-hub/unauthorized-immigrant-population-profiles

©2020 Wilson FA et al. *JAMA Network Open*.

eTable 1. Model Performance Metrics of Random Forest Classifier, Decision Tree Classifier, and Boosted Machine Learning Models Using K-fold Cross-validation, MEPS 2016-2017

	Random Forest		Decision Tree		Boosted	
**Metric**	**Mean (%)**	**SD (%)**	**Mean (%)**	**SD (%)**	**Mean (%)**	**SD (%)**
**Accuracy**	90.5	2.1	89.8	3.1	90.4	2.6
**Specificity**	93.9	2.3	94.1	3.3	94.2	2.8
**Sensitivity**	76.1	6.2	71.2	6.0	73.6	5.3
**Precision**	75.7	4.7	75.4	7.6	75.9	7.0
**F-measure**	75.7	3.6	73.0	5.2	74.5	4.9

©2020 Wilson FA et al. *JAMA Network Open*.

eTable 2. Mean Expenditures per Visit for Respondents Using Health Care by Immigration Status, MEPS 2016-2017

Setting	Unauthorized	Authorized	US-born
**ED**	882.4 (520.4, 1,244.5)	1,142.2 (1,025.9, 1,258.6)	1,090.1 (1,040.1, 1,140.2)
**Inpatient**	3,949.4 (2,565.5, 5,333.4)	5,288.5 (4,622.7, 5,954.3)	5,141.8 (4,835.9, 5,447.7)
**Outpatient**	767.1 (423.3, 1,110.9)	1,156.3 (900.2, 1,412.4)	1,194.9 (1,119.8, 1,269.9)
**Office-based**	183.9 (160.1, 207.7)	238.0 (223.5, 252.5)	239.1 (233.6, 244.6)

MEPS, Medical Expenditure Panel Survey; ED, Emergency Department; CI, Confidence Interval

©2020 Wilson FA et al. *JAMA Network Open*.

eTable 3. Unweighted and Weighted Numbers and Estimated Aggregate National Health Care Expenditures for Each Immigrant Group

Group	Unweighted N	Weighted N*	Percent	Aggregate National Expenditures In 2017
**US-born**	35,877	204,395,952	82.6	$1.3 trillion
**Authorized**	10,927	40,130,940	16.2	$155.8 billion
**Unauthorized**	1,240	2,982,287	1.2	$4.2 billion
**Total**	48,044	247,509,179	100	$1.4 trillion

* The total weighted N compares to the US Census 2019 estimate of 250.56 million civilian noninstitutionalized population in 2019. There are an estimated 44.7 million foreign-born using US Census Bureau American Community Survey estimates for 2019; this compares to 43.1 million in 2017 in our analysis.

©2020 Wilson FA et al. *JAMA Network Open*.

eTable 4. Generalized Linear Model Adjusted Total Health Care Expenditures by Immigration Status

	Expenditures (95%CI)	Difference with Unauthorized
**Unauthorized**	4,105 (3,094, 5,115)	
**Authorized**	4,622 (4,288, 4,955	517
**US-born**	5,490 (5,338, 5,641)	1,385

©2020 Wilson FA et al. *JAMA Network Open*.
